# Lit-OTAR framework for extracting biological evidences from literature

**DOI:** 10.1093/bioinformatics/btaf113

**Published:** 2025-03-17

**Authors:** Santosh Tirunagari, Shyamasree Saha, Aravind Venkatesan, Daniel Suveges, Miguel Carmona, Annalisa Buniello, David Ochoa, Johanna McEntyre, Ellen McDonagh, Melissa Harrison

**Affiliations:** Literature Services Team, European Bioinformatics Institute, European Molecular Biology Laboratory (EMBL-EBI), Wellcome Trust Genome Campus, Cambridge CB10 1SD, United Kingdom; Literature Services Team, European Bioinformatics Institute, European Molecular Biology Laboratory (EMBL-EBI), Wellcome Trust Genome Campus, Cambridge CB10 1SD, United Kingdom; Literature Services Team, European Bioinformatics Institute, European Molecular Biology Laboratory (EMBL-EBI), Wellcome Trust Genome Campus, Cambridge CB10 1SD, United Kingdom; Open Targets, European Bioinformatics Institute, European Molecular Biology Laboratory (EMBL-EBI), Wellcome Trust Genome Campus, Cambridge CB10 1SD, United Kingdom; Open Targets, European Bioinformatics Institute, European Molecular Biology Laboratory (EMBL-EBI), Wellcome Trust Genome Campus, Cambridge CB10 1SD, United Kingdom; Open Targets, European Bioinformatics Institute, European Molecular Biology Laboratory (EMBL-EBI), Wellcome Trust Genome Campus, Cambridge CB10 1SD, United Kingdom; Open Targets, European Bioinformatics Institute, European Molecular Biology Laboratory (EMBL-EBI), Wellcome Trust Genome Campus, Cambridge CB10 1SD, United Kingdom; Literature Services Team, European Bioinformatics Institute, European Molecular Biology Laboratory (EMBL-EBI), Wellcome Trust Genome Campus, Cambridge CB10 1SD, United Kingdom; Open Targets, European Bioinformatics Institute, European Molecular Biology Laboratory (EMBL-EBI), Wellcome Trust Genome Campus, Cambridge CB10 1SD, United Kingdom; Literature Services Team, European Bioinformatics Institute, European Molecular Biology Laboratory (EMBL-EBI), Wellcome Trust Genome Campus, Cambridge CB10 1SD, United Kingdom

## Abstract

**Summary:**

The lit-OTAR framework, developed through a collaboration between Europe PMC and Open Targets, leverages deep learning to revolutionize drug discovery by extracting evidence from scientific literature for drug target identification and validation. This novel framework combines named entity recognition for identifying gene/protein (target), disease, organism, and chemical/drug within scientific texts, and entity normalization to map these entities to databases like Ensembl, Experimental Factor Ontology, and ChEMBL. Continuously operational, it has processed over 39 million abstracts and 4.5 million full-text articles and preprints to date, identifying more than 48.5 million unique associations that significantly help accelerate the drug discovery process and scientific research >29.9 m distinct target–disease, 11.8 m distinct target–drug, and 8.3 m distinct disease–drug relationships.

**Availability and implementation:**

The results are accessible through Europe PMC’s SciLite web app (https://europepmc.org/) and its annotations API (https://europepmc.org/annotationsapi), as well as via the Open Targets Platform (https://platform.opentargets.org/). The daily pipeline is available at https://github.com/ML4LitS/otar-maintenance, and the Open Targets ETL processes are available at https://github.com/opentargets.

## 1 Introduction

The process of identifying drug targets is a critical aspect of drug discovery, requiring an understanding of the molecular and genetic mechanisms of underlying diseases. In this study, a ‘target’ specifically refers to genes or proteins that are investigated for their potential role in disease association and drug discovery. Scientists rely on various sources of evidence such as gene expression changes, genetic variations, and clinical study data to unravel the connections between drugs, targets, and diseases (Kafkas *et al.* 2017). To navigate this complexity, the Open Targets Platform (Buniello *et al.* 2025) was developed as a comprehensive web-based tool that integrates diverse sources of evidence, facilitating the efficient identification of promising drug targets associated with diseases and phenotypes. The Platform combines data from >20 different sources to provide target–disease associations, including evidence derived from genetic associations, somatic mutations, known drugs, differential expression, animal models, pathways and systems biology, and text-mining of scientific articles. An integrated score weighs the evidence from each source and type, contributing to an overall score for each target–disease association. This systematic approach harmonizes information into a coherent schema and presents it in a user-friendly manner.

Extraction of assertions from scientific articles is an important aspect of this work, and to this end, Europe PMC (Rosonovski *et al.* 2024) has played a key supporting role. Europe PMC, a global free biomedical literature repository indexing over 41 million abstracts and 8.7 million full-text articles, provides essential support with its text-mining capabilities. By integrating Europe PMC’s text-mined annotations, the Open Targets Platform harnesses scientific literature as a unique source of information, particularly to identify and elucidate target–disease–drug associations, which are central to its functionality.

**Figure 1.**
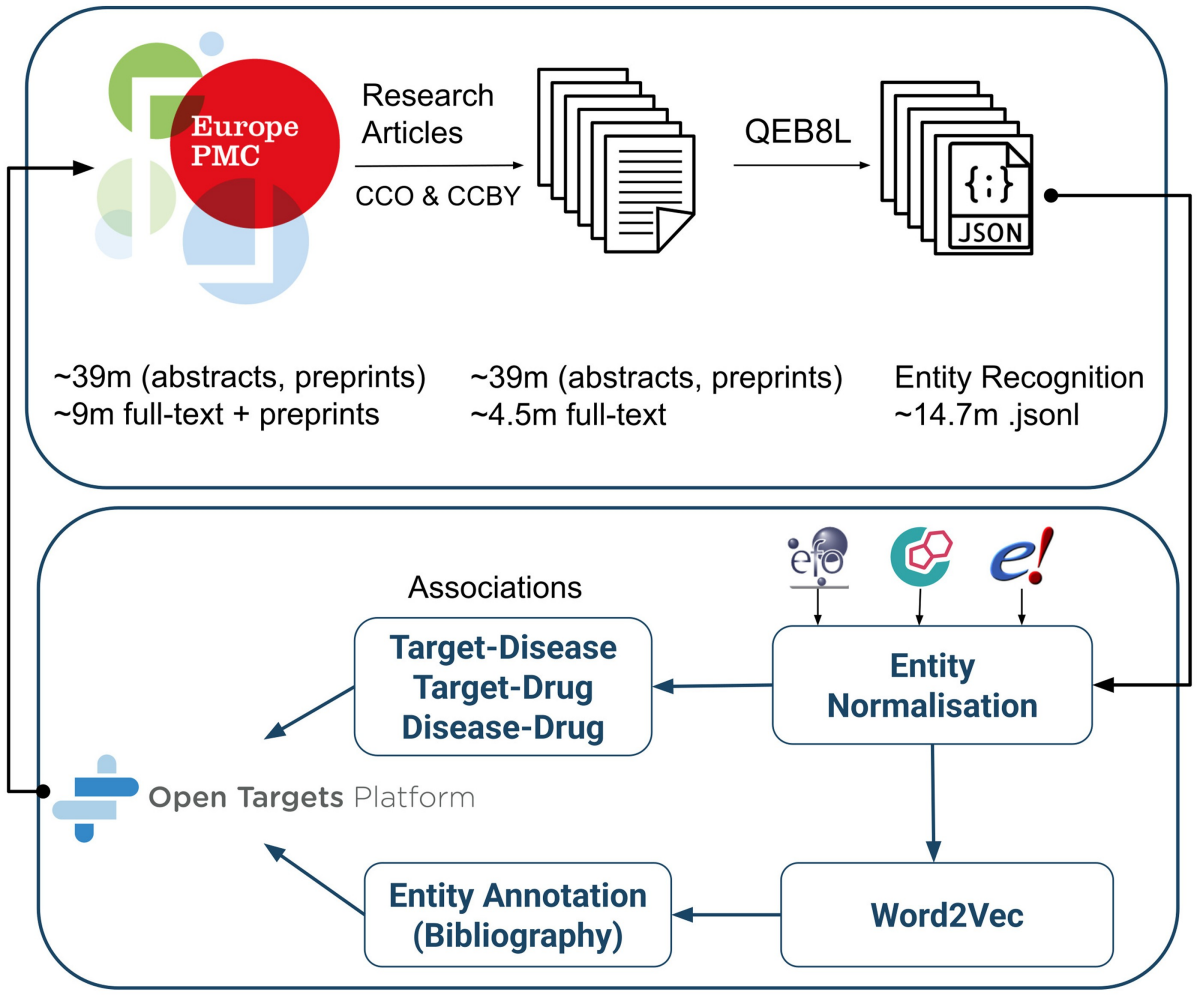
Overview of the data selection, processing, and accessibility workflow in Europe PMC and Open Targets Platforms. Refer to Section 2.1, Section 2.2, and Supplementary Section S4 of the Supplementary Material S7 (Entity Annotation/Bibliography).

The Literature-Open Targets (Lit-OTAR) framework consists of two primary components: Europe PMC text-mining and the Open Targets literature module, as illustrated in Fig. 1. Europe PMC utilizes deep learning techniques to identify target (gene/protein), disease, and chemical/drug entities within scientific documents. Subsequently, Open Targets performs entity normalization to accurately map these entities to databases like Ensembl (Dyer *et al.* 2025), Experimental Factor Ontology (EFO) (Malone *et al.* 2010), and ChEMBL (Zdrazil *et al.* 2024), while ranking the associations between target–disease–drug mentioned in these documents. The primary goal of this framework is to provide a scalable and continuous service to the scientific community, enabling efficient target validation.

Within the existing landscape in biomedical text-mining there are a number of tools focusing on extracting key entities and associations from literature. For instance, DisGeNET (Piñero *et al.* 2020), SemMedDB (Kilicoglu *et al.* 2012), LitSense (Allot *et al.* 2019), PubTator (Wei *et al.* 2024), and PubTator Central (Wei *et al.* 2019) provide efficient ways to access high-quality text-mined bio-entites. However, these resources are oriented towards access to texmined outputs alone either via highlighting terms or via APIs. The Lit-OTAR work described in here is differently oriented, where the outputs of the framework are mainly integrated with other types of evidences (e.g. RNA expression and pathway analysis) to support systematic identification and prioritization of therapeutic drug targets in the Open Targets Platform (Ochoa *et al.* 2023, Buniello *et al.* 2025). The outputs are highlighted using Europe PMC’s Scilite tool and accessed through the Annotations API.

The Lit-OTAR framework also benefits from an active community that provides documentation, training, and feedback to drive continuous improvements. Community collaboration is facilitated through resources such as the Open Targets Community Portal (https://community.opentargets.org) and the European PMC’s developer forum (https://groups.google.com/a/ebi.ac.uk/g/epmc-webservices), where users share insights and updates.

Our work builds on previous efforts. In our 2017 study (Kafkas *et al.* 2017), we used dictionary-based methods within a quarterly operational pipeline for data updates, utilizing the Europe PMC text-mining pipeline enhanced with custom dictionaries from UniProt and EFO for annotating target and disease names. Although this approach was robust, it faced limitations due to its reliance on manual rules such as abbreviation filters and blacklists of common terms and challenges inherent in biomedical texts. Distinguishing between gene and protein names, spelling variations, and context-specific meanings of abbreviations often led to high recall but low precision (Yang *et al.* 2023).

The emergence of modern natural language processing (NLP) techniques (Dang *et al.* 2018, Li *et al.* 2022) has revolutionized text-mining by offering high efficiency and accuracy. Models like BERT (Devlin *et al.* 2018), BioBERT (Lee *et al.* 2020), PubMedBERT (Gu *et al.* 2022), and BioFormer (Fang *et al.* 2023), trained on extensive biomedical corpora and fine-tuned for specific tasks, have markedly improved the accuracy of entity recognition, managing ambiguities, special characters, acronyms, and identifying synonyms and variations in expression. These advancements not only enhance recall and precision rates but also facilitate the discovery of new biological relationships from the extensive, unstructured data in the life science domain.

In our current study, we have leveraged deep learning techniques, specifically models such as BioBERT and BioFormer, to significantly enhance our pipeline. This updated Lit-OTAR pipeline has been refined to enhance flexibility and modularity, expanding its scope to include a new entity category for chemical/drug. This enhancement has enabled the pipeline to text-mine for associations between drugs and targets, drugs and diseases, in addition to targets and diseases. Furthermore, we have addressed technical challenges such as sentence splitting and boundary detection in complex document structures like tables and figures. The main distinctions between the previous and the current pipeline are detailed in Supplementary Table S1 of the Supplementary Material.

## 2 Methods

At the time of writing this article, Europe PMC hosted approximately 39 million journal and preprint abstracts and 9 million full-text journal and preprint articles. However, only a subset of these, specifically 39 million and 4.5 million, respectively, were included due to licensing restrictions (CCO and CC-BY) and their classification as original research articles (https://europepmc.org/Copyright). This dataset and the subsequent daily addition of the data is run through our custom-developed deep learning model for named entity recognition (NER) extraction [QEB8L (https://github.com/ML4LitS/annotation_models)]. The generated output is formatted in JSON, with identified entities treated as matches. Moreover, when two matches or entities occur within the same sentence, they are considered as forming an association or providing evidence. We have completed a study with three experts for treating co-occurrence as association (refer to Section 2.3). Subsequently, this processed data is forwarded to the Open Targets ETL for the purpose of normalization (grounding). Disease-related entities are mapped to the EFO, chemical and drug entities to CHEMBL, and gene and protein entities to Ensembl. The resulting data is made accessible through both the Open Targets Platform and Europe PMC annotations APIs, in addition to the Scilite annotations tool (Venkatesan *et al.* 2016) on the Europe PMC website (refer to Supplementary Material S7: Supplementary Section S2).

### 2.1 Entity recognition

To develop deep learning models for the Lit-OTAR framework, we utilized the Europe PMC dataset (Yang *et al.* 2023). Initially, this dataset did not include mentions of chemical/drug. To overcome this limitation, we used CHEMDNER BioCreative dataset (Krallinger *et al.* 2015) to annotate the corresponding subset in Europe PMC with chemical/drug mentions, preserving the human-annotated spans. The enriched dataset now covers mentions of gene/protein, disease, chemical/drug, and organism. We trained and evaluated three different models BioBERT, SpaCy [custom trained with PubMed+PMC word2vec (Moen and Ananiadou 2013) over 10 iterations], and Bioformer on this dataset, using the evaluation criteria from SemEval-2013 Task 9.1 (Segura-Bedmar *et al.* 2014) (Supplementary Material S2).

### 2.2 Entity normalization

The NER tagging of entities occurs at Europe PMC, while normalization and ranking (Supplementary Material S4) takes place on the Open Targets Platform (Fig. 1).

The process involves matching and mapping entities to specific databases/ontologies. The pipeline uses a Word2Vec skip-gram model (Mikolov *et al.* 2013) to transform NER outputs into standardized representations. This includes mapping diseases to the EFO, chemicals to ChEMBL, and genes to Ensembl. The model generates n-dimensional word embeddings, which capture semantic similarities by analysing co-occurrence patterns in the literature.

The model’s calculation of similarity metrics also supports the ranking of entities, determining their relevance to the research context by analysing literature patterns. This method improves the accuracy of entity normalization and enhances the Open Targets Platform’s value for researchers by offering a comprehensive understanding of biological entity relationships and their potential therapeutic implications (refer to Supplementary Material S3 and Supplementary Algorithm S1).

### 2.3 Co-occurrence versus association

A curation task was conducted to annotate 252 sentences for association analysis, with each annotator pair assigned 168 sentences and an intentional overlap of 84 sentences between pairs to measure inter-annotator agreement. The annotation categories for association included the following classes: Altered Expression, Genetic Variation, Regulatory Modification, Any (general or unspecified association), NA (Not Available), and No (No association mentioned). The annotation overlap was measured using Cohen’s Kappa (K).

Despite high expectations, the overall Cohen’s Kappa value indicated a variance in perceptions of associations in the range of [0.2–0.39], reflecting a low inter-annotator agreement that deemed the overlap unsuitable for machine learning purposes. The association identification presented challenges, evidenced by a low overlap. This difficulty was attributed to various factors, including short sentences lacking clear relations, long sentences with lists of multiple genes/proteins, drugs and diseases, complex sentence structures, and sentences that required additional context for accurate interpretation.

Given the subjectivity in defining associations, we opted to treat co-occurrence as a form of association, including even the absence of explicit associations. This approach allows users to apply post-processing to tailor the data to their specific needs. However, it is important to note that this definition limits the framework’s ability to capture associations that span multiple sentences, such as those involving coreference or inferred context. This constraint may affect the comprehensiveness of extracted associations, as more complex linguistic relationships are challenging to identify.

Following this study, we recognized that association is subjective, leading us to consider co-occurrence as a form of association itself. Consequently, we adjusted our approach to treat co-occurrence as the universal set, acknowledging that any co-occurrence might imply an association, despite the challenges in explicit identification by annotators.

## 3 Results

### 3.1 Entity recognition

BioBERT led in precision among the models tested, achieving scores of 0.91 (Chemical/Drug), 0.90 (Disease), 0.93 (Organism), and 0.91 (Gene/Protein), with similarly high recall and F1-scores, demonstrating its effectiveness in entity recognition across various categories. Given the computational demands of BioBERT, our focus shifted towards enhancing the Bioformer-8L model into the QEB8L model. By utilizing ONNX for model optimization, we significantly improved inference speeds without sacrificing performance. Further enhancements through static quantization not only increased processing speed 10-fold but also reduced the model size to approximately 77MB, all while maintaining impressive accuracy with precision scores ranging from 0.85 to 0.94 and F1-scores around 0.88 to 0.89, highlighting its balanced performance as shown in Table 1.

**Table 1.** Performance of different models across various categories.

Category	Model	Precision	Recall	F1-score
Chemical/drug	Dictionary	0.53	0.34	0.41
	**BioBERT**	**0.91**	**0.92**	**0.92**
	spaCy	0.80	0.73	0.76
	QEB8L	0.85	0.90	0.88
Disease	Dictionary	0.48	0.74	0.58
	BioBERT	**0.90**	0.80	0.85
	spaCy	0.82	0.71	0.76
	**QEB8L**	**0.90**	**0.88**	**0.89**
Organism	Dictionary	0.68	0.90	0.78
	**BioBERT**	0.93	**0.86**	**0.90**
	spaCy	0.85	0.75	0.79
	QEB8L	**0.94**	0.85	0.89
Gene/protein	Dictionary	0.48	0.74	0.58
	**BioBERT**	**0.91**	0.87	**0.89**
	spaCy	0.84	0.76	0.80
	**QEB8L**	0.90	**0.88**	**0.89**

Bold values indicate the best performing model and the highest performance (Precision, Recall, or F1-score) within each category.

SpaCy, recognized for its quick inference speed, presented slightly lower precision scores (0.80–0.84) and F1-scores (0.76–0.80) across categories, suggesting its practicality for production-level entity recognition tasks with its efficiency. Conversely, the Dictionary approach (our previous pipeline), while serving as a baseline in the current study achieved lower precision scores (0.48–0.68) but higher recall in some instances, leading to moderate F1-scores.

**Figure 2.**
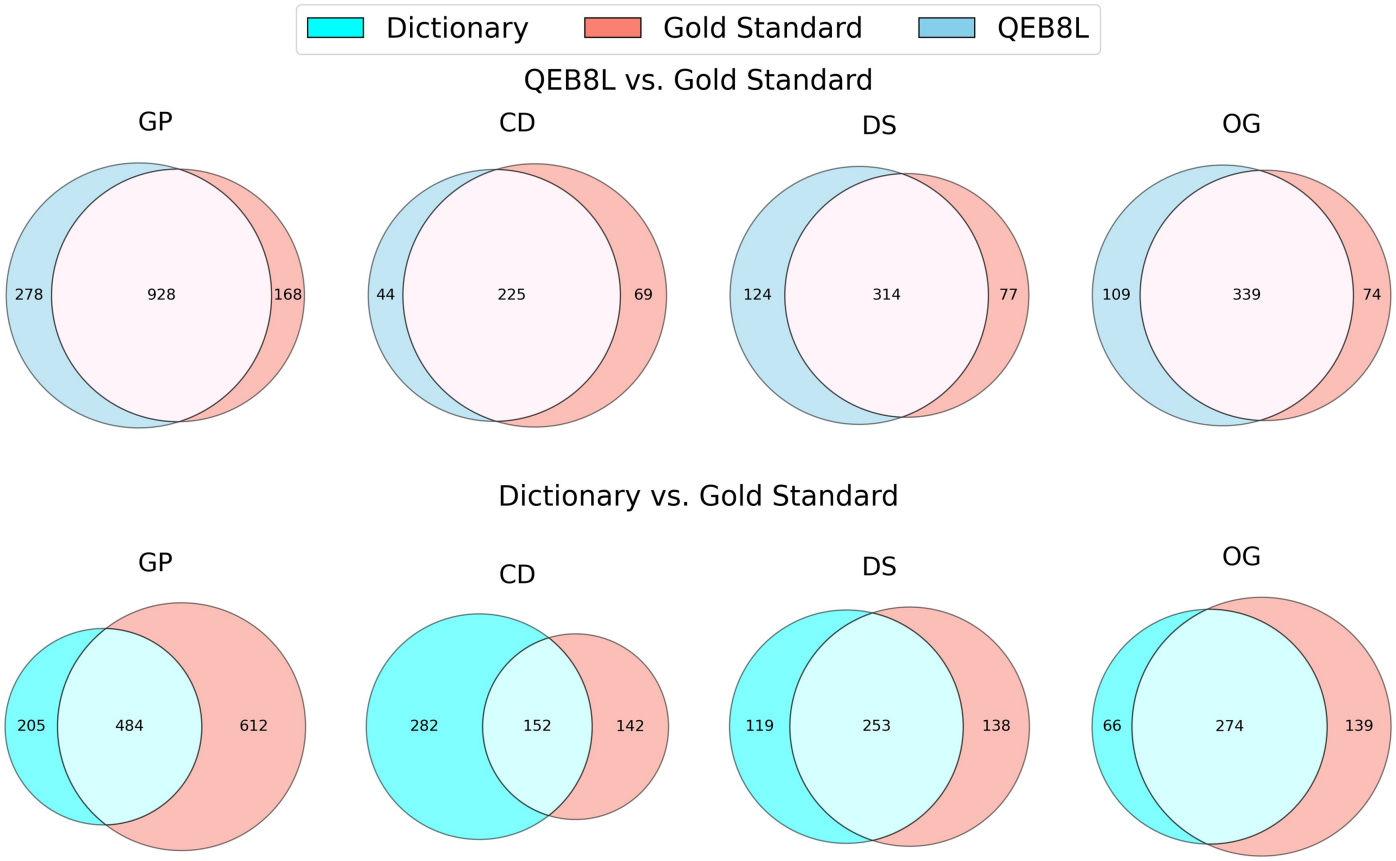
The figure shows the comparison in the number of entity matches between the dictionary-based approach and the gold standard set versus the proposed deep learning approach (QEB8L) to the gold standard (Yang *et al.* 2023). The comparison is made between the entities: Gene/Protein (GP), Chemical/Drug (CD), Disease (DS), and Organism (OG) (refer to Supplementary Material S5).

Our analysis demonstrated a significant overlap between the gold standard and the QEB8L model, identifying additional entities by the QEB8L model not found in the gold standard. Entities identified by the QEB8L model and dictionaries, but absent in the gold standard, were classified as false positives. Our goal was to minimize these false positives while maximizing overlap as illustrated in Fig. 2.

Moving to a deep learning approach, specifically the QEB8L model, was driven by the need to reduce false positives and improve entity coverage. The performance comparison using the gold standard test set demonstrated that the QEB8L model significantly outperformed the previous dictionary-based method, highlighting its advantage. The QEB8L model, trained and evaluated on the gold standard dataset, demonstrated the highest overlap with the gold standard, featuring fewer false positives and false negatives as shown in Fig. 2. Some example entities are explained in Supplementary Material S5 (refer to Supplementary Table S2 for the QEB8L model and Supplementary Table S3 for the dictionary NER approach).

### 3.2 Entity normalization

A large proportion of the recognized entities could be normalized, showing the effectiveness of our methodology in mapping biomedical entities to standardized knowledge bases; Disease to EFO, chemicals to ChEMBL, and genes to Ensembl. This process is crucial for aggregating and analysing biomedical literature, facilitating the identification of relationships between diseases and potential therapeutic targets.

The entity normalized data, as shown in Table 2, illustrates the scale and complexity of biomedical terminology. Diseases and syndromes alone account for over 220 million entities, with approximately 76.6% successfully normalized to known entities from EFO. However, this represents only 7.6% of the unique entity count, highlighting the presence of a long tail of highly heterogeneous and less frequent labels, where rare or variant terms are harder to normalize. The unmapped entities underscore the diversity and complexity of biomedical literature, presenting challenges in achieving complete normalization, yet remain available for further study (https://ftp.ebi.ac.uk/pub/databases/opentargets/platform/latest/output/etl/json/literature/failedCooccurrences/).

**Table 2.** Summary of entity recognition and normalization outcomes across Disease/Syndrome, Chemical/Drug, and Gene/Protein categories.^a^

Entity type	Entity count	Mapped entity count	Unique entity count	Mapped unique entity count	Unique mapped references
Disease	220 392 937	168 818 017 (76.6%)	2 196 439	166 497 (7.6%)	11 561
Chemical/Drug	122 872 756	77 826 420 (63.3%)	2 213 483	76 194 (3.4%)	10 370
Gene/Protein	347 835 641	197 124 445 (56.7%)	7 063 573	680 368 (9.6%)	28 778

a *Entity count* is the total number of entities identified. *Mapped entity count* is the number of these entities normalized to a knowledge base. *Unique entity count* refers to the total distinct entities, while *Mapped unique entity count* is the subset of those distinct entities that were successfully normalized. *Unique mapped references* denote the unique knowledge base identifiers to which entities have been mapped.

The significant diversity among unnormalized entities necessitates continuous refinement of recognition and normalization techniques. A literature-based evidence set curated by the Uniprot team was used to benchmark the performance, focusing particularly on disease-to-target associations. This set, comprising of 969 publications with 1038 disease–target associations, served as a foundation for evaluating the efficiency of the lit-OTAR framework.

The evaluation, detailed in Table 3, demonstrates high match rates for target identification, indicating the Lit-OTAR framework’s potential for mapping biomedical entities to standardized knowledge bases. Conversely, disease recognition and normalization presented less robust results, highlighting areas for improvement due to the complexity and variability of disease nomenclature.

**Table 3.** Benchmarking entity recognition and normalization performance using a UniProt-curated gold standard evidence set.

	Publications	Publication/target pairs	Publication/disease pair	Publication/disease/target triplet	Disease/target pair
Uniprot curated evidence	969	1088	1515	1580	1038
Normalized matches	967 (99.8%)	1034 (95.0%)	1034 (68.3%)	748 (47.3%)	550 (53.0%)

These findings present the strengths and challenges of current Lit-OTAR framework, emphasizing the need for advancements in handling the diversity and complexity of disease terms. Interestingly, Lit-OTAR also facilitated unexpected achievements, including the discovery of new disease entities and synonyms (‘T2D’), and enhanced data processing capabilities by integrating with databases like EFO and improving analyses with the FDA’s Adverse Events Reporting System (FAERS). The details are presented in Supplementary Material S6.

## 4 Conclusions

The Lit-OTAR framework, a collaboration between Europe PMC and Open Targets, harnesses biomedical literature to advance drug discovery. By applying NER and entity normalization, this framework has processed >39 million abstracts and 4.5 million full-text articles, identifying around 48.5 million unique associations among target–disease, target–drug, and disease–drug interactions. This study provides insights into the drug discovery process and expands scientific research. In addition, the framework demonstrates the capability to discover new entities and enrich databases and ontologies with previously unrecognized associations. The Lit-OTAR pipeline operates daily, with updates provided quarterly on both the Europe PMC and Open Targets Platforms, ensuring timely access to relevant data for researchers and supporting therapeutic research and development.

## Supplementary Material

btaf113_Supplementary_Data

## Data Availability

The results generated in this study are accessible through multiple platforms. They can be explored interactively via the Europe PMC SciLite web application (https://europepmc.org/) and the corresponding annotations API (https://europepmc.org/annotationsapi). The data is also integrated into the Open Targets Platform (https://platform.opentargets.org/). For direct access, the latest data releases are available for download via FTP at https://ftp.ebi.ac.uk/pub/databases/opentargets/platform/latest/output/literature/ and can also be queried through Google BigQuery (https://platform-docs.opentargets.org/data-access/google-bigquery). Further technical details and descriptions of the platforms are provided in Supplementary Material S7.
