# Supplementary material for: Lit-OTAR framework for extracting biological evidences from literature
Source: Bioinformatics. 2025 Mar 17;41(4):btaf113. doi: 10.1093/bioinformatics/btaf113 (PMC11978389; doi:10.1093/bioinformatics/btaf113)
Supplement: btaf113_Supplementary_Data [file btaf113_supplementary_data.pdf]

Supplementary Material

Article Title: Lit-OTAR Framework for Extracting Biological Evidences from Literature

Authors: Santosh Tirunagari, Shyamasree Saha, Aravind Venkatesan, Daniel Suveges, Miguel Carmona, Annalisa Buniello, David Ochoa, Johanna McEntyre, Ellen McDonagh, and Melissa Harrison
Affiliation: European Bioinformatics Institute, European Molecular Biology Laboratory (EMBL-EBI), Wellcome Trust Genome Campus, Hinxton, CB10 1SD, Cambridge, United Kingdom

S1: Old pipeline vs new Lit-OTAR pipeline

Aspect	Old pipeline [4]	New Lit-OTAR pipeline
Data Source	Europe PMC (PubMed and PubMed Central) CCO & CC-BY Original Research articles	Europe PMC (PubMed and PubMed Central, and Preprints). CCO & CC-BY Original Research articles
Data Size	26 million abstracts, 1.2 million full-text articles	39 million journal article and preprint abstracts and 4.5 million full-text articles and counting
Approach	Dictionary-based	Deep learning (Bioformer-8L)
Types	Genes/protein and Disease	Genes/protein, Disease, Organisms, and Chemical/Drug
Accuracy	High recall but low precision	Improved precision and recall
Evidences	Gene–Disease	Gene–Disease<br>Gene–Drug<br>Disease–Drug
Article Scoring	Confidence scores based on location	Confidence scores based on location (similar to old pipeline (1))
Operational	Quarterly (terminated on 04/2021)	Daily since 04/2021
Benchmarking	None	Benchmarking of NER methods
Notes	Manual rules, abbreviation filter with heuristic rules, limited completeness, false positives	Improved accuracy, normalisation, broader scope, improved sentence splitter, reduced limitations
Performance	Precision: 0.54<br>Recall: 0.67<br>F-score: 0.58	Precision: 0.90<br>Recall: 0.88<br>F-score: 0.89

Table T1. Comparative Analysis of the Old pipeline (1) and the Current pipeline Across Various Aspects

DRAFT

S2: Evaluation Criteria

The NER was evaluated using the evaluation criteria used by SemEval-2013 Task 9.1 (24), which allows assessment of the system's performance based on four levels of strictness: strict, exact, partial, and type. Strict evaluation requires both the boundaries and the type of an entity to match exactly with the reference annotation, meaning even slight boundary differences result in a mismatch. Exact evaluation, on the other hand, focuses on the boundaries alone, ensuring they match perfectly without factoring in type accuracy. These levels consider the match of entity boundaries and types. Using these metrics to evaluate the performance of the NER go beyond simple strict classification and take into account partial matching. To compare the differences between the output of the NER system and the correct annotations, two factors were considered: the exact string and the type of the entity. However, because there can be overlapping entities from different categories and data formats, each system per category was evaluated. This means that in certain cases, the counts in the "Strict" and "Exact" cells become equal. Similarly, this applies to the values in the cells that correspond to partial matching and incorrect matching. After evaluating the NER system using the metrics discussed above, the precision, recall, and F1-score was calculated for benchmarking.

The entity-level **precision** and **recall** are computed by deciding when a predicted entity counts as a correct match (COR), in contrast to being labeled as partial (PAR), incorrect (INC), spurious (SPU), or missed (MIS). Once "correct" is defined under a particular matching scheme (Strict, Exact, Partial, or Type), we use the usual formulas[8]:

$$\text{precision} = \frac{\text{correct}}{\text{actual}}, \qquad \text{recall} = \frac{\text{correct}}{\text{possible}},$$

where

$$\text{actual} = \text{number of system output entities (TP + FP)}, \quad \text{possible} = \text{number of gold (true) entities (TP + FN)}.$$

Match Schemes in Detail.

1. **Strict.** A system entity is counted as correct (COR) only if:
   - Its boundaries match the gold entity exactly (same start and end tokens),
   - and the type is identical (e.g., both are `DISEASE`).

   If either boundary or type differs, it is labeled as INC (incorrect), PAR (partial), etc.
2. **Exact.** The boundaries must match exactly, but the entity type is ignored for correctness. Thus, a perfect boundary match is always COR, regardless of the predicted vs. gold type.
3. **Partial.** Any overlap between a system-predicted entity and a gold entity is at least a partial match. Following Batista's implementation:

   $$\text{COR}_{\text{partial}} = \text{(full overlaps)}, \quad \text{PAR}_{\text{partial}} = \text{(partial overlaps)}.$$

   Partial matches contribute half a point in precision and recall:

   $$\text{precision}_{\text{partial}} = \frac{\text{COR} + 0.5 \times \text{PAR}}{\text{actual}}, \quad \text{recall}_{\text{partial}} = \frac{\text{COR} + 0.5 \times \text{PAR}}{\text{possible}}.$$

   Types are ignored in the "Partial" scheme.
4. **Type.** The system entity must overlap with the gold entity and must match its type. Overlaps with the same type are counted as COR (for full overlap) or PAR (for partial). Partial overlaps receive half credit, while overlaps with different types are INC.

Finally, each system entity is ultimately labeled as one of five:

- **COR** – correct
- **INC** – incorrect
- **PAR** – partial match
- **MIS** – missed (the gold entity was not found)
- **SPU** – spurious (the system predicted an entity that does not exist in gold)

Then, for each of the four schemes (Strict, Exact, Partial, Type), we decide what qualifies as "COR." In the Partial and Type evaluations, partial matches (PAR) count as 0.5 toward precision and recall. Finally, the precision, recall, and F1-score are computed for each of these schemes to benchmark system performance.

DRAFT

[8] http://www.davidsbatista.net/blog/2018/05/09/Named_Entity_Evaluation/

S3: Entity Linking

The entity linking process, implemented in Scala and integrated into the Open Targets pipelines https://github.com/opentargets/platform-etl-backend/blob/master/src/main/scala/io/opentargets/etl/backend/literature/, is designed to efficiently map disease, drug, and gene labels to their corresponding EFO, ChEMBL, or Ensembl identifiers. This approach involves generating a comprehensive lookup table from the Open Targets Platform's disease, target, and drug indices. Each dataset is processed independently, with all possible labels-such as names, symbols, and retired terms-being expanded for each identifier. To rank the mapped keywords, similarity factor is applied, prioritizing, for example, approved symbols over obsolete names. In cases where a single label maps to multiple identifiers with equal ranking, the process disambiguates by aggregating all related labels and identifiers from the source paper, selecting the most representative identifier based on the assumption that multiple mentions of the same entity in a paper will use various synonyms. This ensures accurate linking and minimizes redundant matches in the evidence.

The Algorithm A1 outlines a structured approach starting with the "Main" Procedure, where the initial data comprising entity matches (named entities), and sentence texts is loaded and passed through various stages. Note that in Europe PMC pipeline, only sentence splitting and section tagging are performed; the sentences are then fed directly to the QEB8L model for entity recognition based on the subword tokenization provided by Bioformer. By contrast, the Open Targets pipeline (Algorithm A1) carries out its own NLP preprocessing.

In the first stage PreprocessData, the data undergoes filtering based on entity types and publication sections. This function also normalizes the text to ensure uniformity (UTF-8 conversion).

Next, the GroundEntities function applies NLP techniques such as tokenization, stopword removal, and stemming. These steps help in breaking down the text into manageable units and prepare the data for entity linking. Following this, the Word2VecModel function trains a Word2Vec model on the preprocessed text, using parameters like window size and iteration count to guide the training process. This trained model serves as the basis for mapping text to entities.

In the MapTextToEntities function, the trained Word2Vec model is applied to map text data to corresponding entities by measuring similarity. A similarity threshold (70%) is employed to determine valid matches, and the function adjusts mappings to optimize performance. Post-processing is handled by the PostProcessOutput function, which resolves co-occurrences and ranks the results based on defined metrics, ensuring that the output is both relevant and accurate.

Finally, the entity mappings, co-occurrence information, and failed mappings are saved to specified output paths (SaveOutput function). This structured pipeline ensures that the NER data is effectively processed, entity linked, and saved for further analysis or reporting.

DRAFT

S4: Article Scoring and Ranking

The article scoring method in the lit-OTAR framework follows the approach detailed in (1). The algorithm scores scientific articles on their relevance to target–disease associations, helping to rank/prioritise articles by their relevance. The algorithm uses a weighting system that assigns different values to article sections, from full-text articles to abstracts, based on their ability to highlight key entities. For instance, the "Title" section gets the highest weight as it summarises the study's findings, while the "Introduction" is weighted least, given its focus is on known information. In abstracts, weight is given based on sentence position, with the analysis of 360 MEDLINE abstracts (1) showing that the last sentence, usually detailing results, is considered most significant.

S5: Examples: False Positives and False Negatives

The examples of entities found through QEB8L but missed in the Gold Standard (False Positives) versus entities found in the Gold Standard but missed through QEB8L (False Negatives) are presented in

Algorithm A1 Entity Linking Pipeline

```
Input: Matches, entities (diseases, drugs, targets), document texts (abstracts, full texts)
Output: Entity mappings, Word2Vec vectors, co-occurrence data
procedure MAIN
    Load data: matches, entities, texts                          ▷ Assuming data is pre-structured
    PreprocessData()
    GroundEntities()
    Model ← Word2VecModel()
    EntityMappings ← MapTextToEntities(Model)
    PostProcessOutput(EntityMappings)
    SaveOutput()
end procedure
function PREPROCESSDATA                                           ▷ Filter and organize initial datasets for processing
    Apply filters based on entity types and sections
    Normalize text data for uniformity
    return preprocessed data
end function
function GROUNDENTITIES                                           ▷ Apply NLP techniques to identify and normalize entities
    Tokenize text to separate words
    Remove stopwords and apply stemming
    Prepare NLP pipelines for data transformation
    return grounded entities
end function
function WORD2VECMODEL                                            ▷ Train a Word2Vec model using the preprocessed text
    Configure model parameters (window size, etc.)
    Train model on organized text data
    return trained model
end function
function MAPTEXTTOENTITIES(Model)                                 ▷ Map text to entities using a trained Word2Vec model
    Apply model to text data
    Use a similarity threshold to determine entity matches
    Adjust mapping based on performance
    return mappings
end function
function POSTPROCESSOUTPUT(Mappings)                              ▷ Resolve and refine entity mappings and relationships
    Analyze co-occurrence and contextual data
    Rank and merge results based on defined metrics
    return refined output
end function
function SAVEOUTPUT                                               ▷ Persist the final output data for analysis or reporting
    Configure paths and formats for saving data
    Save EntityMappings and co-occurrence data
end function
```

DRAFT

Table T2. Similarly, Table T3 presents entities found through the Dictionary Approach but missed in the Gold Standard (False Positives) versus entities found in the Gold Standard but missed through the Dictionary Approach (False Negatives).

Entity Type	Found in QEB8L but missed in Gold Standard	Found in Gold Standard but missed in QEB8L
**Gene/Protein (GP)**	[’CPZ’, ’Flp’, ’APP180’, ’hALK’, ’CT4’]	[’YALI0D20108g’, ’YALI0E32901g’, ’LDH4’, ’YALI0A9470g’, ’MSC1’]
**Chemical/Drug (CD)**	[’No916601429’, ’phycoerythrin’, ’thymidine’, ’butyrate’, ’crystal’]	[’YALIOB19470’, ’11192732’, ’pentose phosphate’, ’QAPP67’, ’CS36962’]
**Disease (DS)**	[’ischemia’, ’CUMS’, ’CIS’, ’facial angiofibromas’, ’Rhizoma’]	[’aneurysms’, ’hallucinations’, ’Postherpetic Neuralgia’, ’lymphadenopathy’, ’pertussis’]
**Organism (OG)**	[’Platyhelminthes’, ’protozoans’, ’merozoites’, ’SZ’, ’kids’]	[’Murine’, ’Methanobrevibactr’, ’wisent’, ’proviruses’, ’hermaphrodite’]

Table T2. Example Entities Found through QEB8L but Missed in Gold Standard vs. Entities Found in Gold Standard but Missed through QEB8L.

For instance, in Table T3, the organism “cotton" was missed in the Gold Standard. The term “cotton" in the given context refers to bedding material rather than the plant species, as shown in the sentence:

> Each male compartment contained a stainless steel nest-box (130 mm × 130 mm × 130 mm) filled with cotton bedding, a cardboard tube, water bowl, feed tray, and plastic climbing lattice on one wall. (PMCID: PMC4414469, Figure 1)

This differs from its occurrence in the Gold Standard, where “cotton" refers to the plant in an agricultural context:

> Geminiviruses are emerging plant pathogens that infect a wide variety of crops including cotton, cassava, vegetables, ornamental plants, and cereals. (PMCID: PMC3024232, Section Abstract)

Similarly, the chemical entity term “sec" was tagged in one context as referring to "seconds" rather than the intended chemical meaning. Additionally, the term "hermaphrodite" (an organism) was confused with “hermaphroditism", which may be considered a disorder in certain contexts.
These examples highlight the limitations of the dictionary-based NER approach used to extract entities. While this approach relies on predefined dictionaries and manual rules, such as abbreviation filters and blacklists of common terms, it faces challenges in handling the complexity and variability inherent in biomedical texts. Issues such as distinguishing between gene and protein names (e.g., p53 vs. P53), managing spelling variations (e.g., T2D vs. T2DM), and interpreting context-specific meanings of abbreviations (e.g., AIDS vs. aids) can lead to errors. The example of “cotton," as discussed earlier, underscores the difficulty in disambiguating context-specific meanings. Additionally, special characters, synonyms, and variations in word choice and sentence structure further complicate entity recognition, often necessitating human interpretation and an exhaustive list of dictionary terms. As a result, this approach, while achieving high recall, often suffers from low precision.
Entities found by QEB8L but missed in the Gold Standard (Table T2) include “merozoites" (PMCID: PMC3097211), which are small, egg-shaped, unicellular organisms that represent a motile stage in the life cycle of malaria parasites:

> After intense multiplication during 2–6 days, depending on the Plasmodium species, mature EEFs release thousands of merozoites, which invade erythrocytes and initiate the pathogenic blood stage cycle.

This was not annotated in the Gold Standard but was correctly identified as an organism by the QEB8L model.
However, there were also instances of misidentification. For example, “Chronic Unpredictable Mild Stress (CUMS)" (PMCID: PMC4931053) was incorrectly identified as a disease, whereas it actually refers to an

DRAFT

Entity Type	Found in Dictionary approach but missed in Gold Standard	Found in Gold Standard but missed in Dictionary approach
**Gene/Protein (GP)**	['nodal', 'Calc', 'MPI', 'LPS', 'NHLT']	['mTau', 'At1g61795', 'eIF2', 'proton / Pi symporters', 'collagen type IV']
**Chemical/Drug (CD)**	['3At', 'silver', 'sec', 'Peptide', 'Lipopolysaccharide']	['nucleotide', 'YALIOB19470', 'carboxylates1617', 'serine', '11192732']
**Disease (DS)**	['Trauma', 'ischemia', 'facial angiofibromas', 'bluetongue', 'hermaphrodite']	['CHD', 'SZ', 'RR - MS', 'B - NHL', 'memory deficits']
**Organism (OG)**	['Euglenozoa', 'Platyhelminthes', 'cotton', 'Białowieża', 'Gibbon']	['Gram - positive cocci', 'bulls', 'Euglenezoa', 'Methanobrevibactr', 'rodent']

Table T3. Example Entities Found through Dictionary Approach but Missed in Gold Standard vs. Entities Found in Gold Standard but Missed Through Dictionary Approach.

experimental method. Similarly, in another context (PMCID: PMC5528876), "CIS" (Checklist Individual Strength) was tagged as a disease, likely due to confusion with "clinically isolated syndrome".
Given that the QEB8L model tends to tag numerous spurious terms, it is crucial to normalize these terms to a knowledge base using entity linking.

S6: Other Achievements

The additional entities not found in dictionaries or the gold standard test set (Figure 2), which have been discovered through context learning in deep learning, further facilitate the addition of new entities to databases/ontologies. In one such scenario, the framework has aided in identifying diseases previously unlinked to any specific disease entity within the EFO [9]. Through a preliminary analysis of frequently occurring non-grounded labels, we identified new synonyms for existing diseases, notably recognising "T2D" as a synonym for Type II Diabetes Mellitus ($EFO\_0001360$). This discovery alone added 281,184 matched labels across $29,040$ unique PubMed identifiers (PMIDs), significantly enriching the dataset and enhancing the accuracy of disease-related data mapping.
In another scenario, it enhanced the processing of data from the FDA's Adverse Events Reporting System (FAERS). This system, which compiles reports of adverse events and medication errors submitted to the FDA, presents unique challenges, such as distinguishing between a drug's adverse events and its indications. To address this, an increase in EFO cross-references to MedDRA was necessary in order to find out whether excluding reports where the adverse event matches the drug indication could improve the analytical outcomes' power and effectiveness. The normalisation pipeline developed for the Lit-OTAR was able to map a significant portion of MedDRA[10] labels associated with adverse reactions to their corresponding EFO terms. This effort resulted in a cross-reference list containing approximately 10,000 mappings, which are assessed to be highly reliable.

DRAFT

S7: Data Platforms

The datasets generated are made available through both Open Targets Platform and Europe PMC. While Open Targets Platform provides a web interface for data exploration and as bulk download, in Europe PMC the datasets are accessible both via the Annotations API and the website.

A. Europe PMC Annotations API. The Europe PMC Annotations API[11] is one of the main methods of accessing texted mined outputs (also called annotations) hosted by Europe PMC. Derived from both abstracts and open access full-text articles, these annotations are an invaluable resource for researchers needing programmatic access to the vast repository. One of the motivations of this is to make text-mined annotations available to the larger scientific community. To this end, the annotations are modelled based

[9] https://github.com/opentargets/issues/issues/1555?ref=blog.opentargets.org?ref=blog.opentargets.org
[10] https://www.meddra.org/?ref=blog.opentargets.org
[11] www.europepmc.org/AnnotationsApi

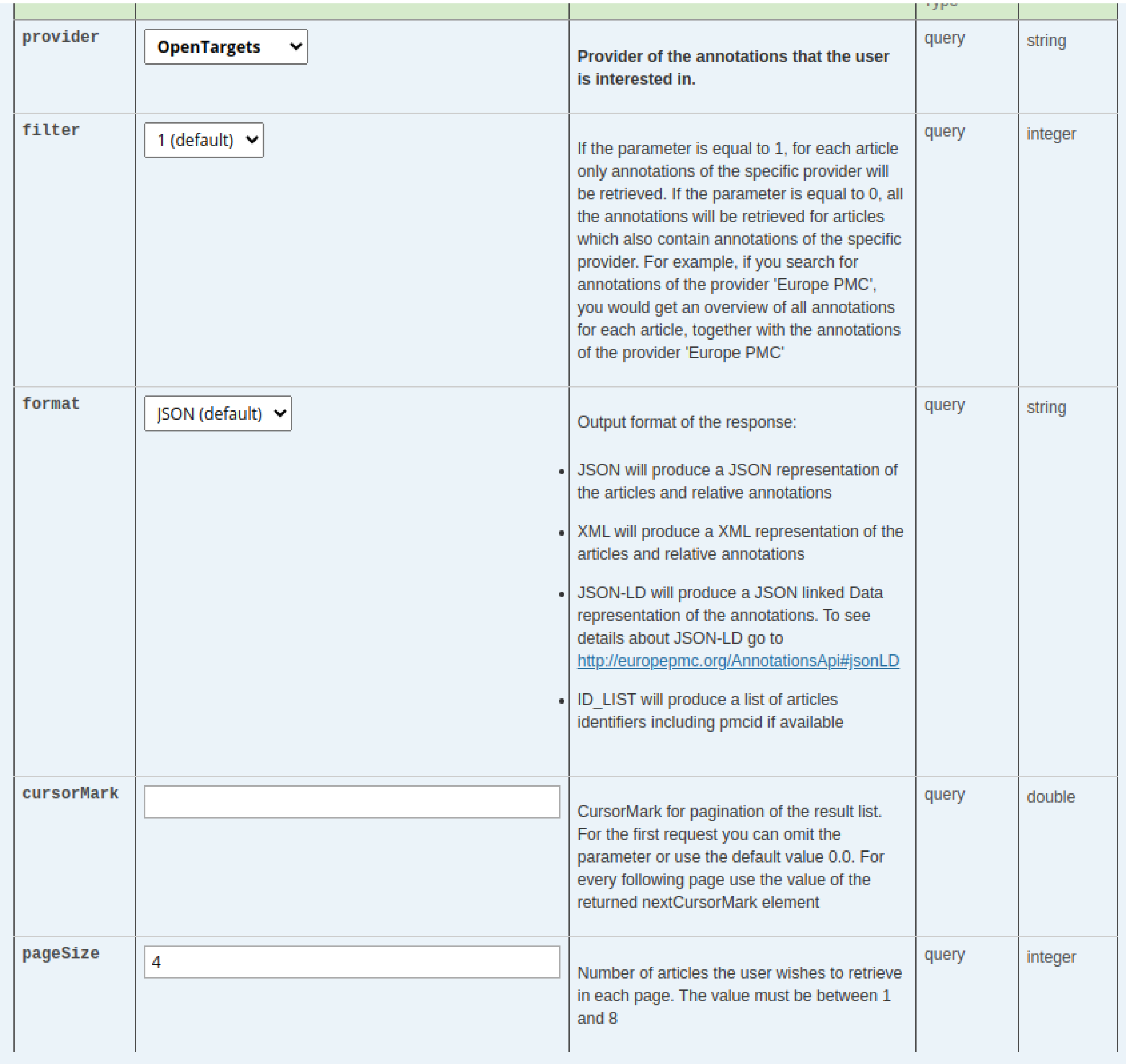

Fig. F1. Accessing annotations by OpenTargets on the Europe PMC annotations API

on the W3C Web Annotation Data Model[12]. This has ensured the annotations are standardised and, most importantly, FAIRified for wider consumption. The annotations are made available under the Apache License Version 2.0[13].

The API's RESTful architecture offers a modular structure, facilitating various functionalities essential for fetching specific annotations based on article IDs, entities, providers, relationships, or article sections. This flexibility is crucial for researchers aiming to extract detailed and targeted information from the literature. The functionality varies from fetching annotations by article, entity name and provider (e.g. OpenTargets) to relationships such as gene-disease relationships (see Figure F1).

The API delivers results in various formats, including JSON, XML, and ID_LIST (for article identifiers), catering to different user preferences and requirements. Moreover, the annotations are available in JSON-LD format, providing a graph representation that enhances data interoperability and integration.

[12] https://www.w3.org/TR/annotation-model/
[13] https://www.apache.org/licenses/LICENSE-2.0

This extensive accessibility to annotated biomedical literature through the Europe PMC Annotations API significantly empowers researchers, pharmaceutical companies facilitating the extraction and analysis of rich datasets for advancing scientific discoveries.

B. Visualisations on Europe PMC Website. SciLite (21) is an annotation tool integrated into Europe PMC that highlights key biological concepts within scientific articles. SciLite enables researchers to quickly grasp the important elements of a paper, facilitating more efficient data discovery and making it easier to cross-reference information. The application makes API requests using the Annotations API to fetch all relevant annotations for a given article (see Figure F3). A detailed description of the design behind SciLite can be found here.[14] SciLite is one of the infrastructural components of the Europe PMC annotation platform. The platform is open for text-mined outputs from any source to be shared and displayed seamlessly on content. This is enabled by the use of the (W3C recommended) Web Annotation Data Model `http://www.w3.org/TR/annotation-model/`. This aspect differentiates SciLite from other tools such as PubTator (10).

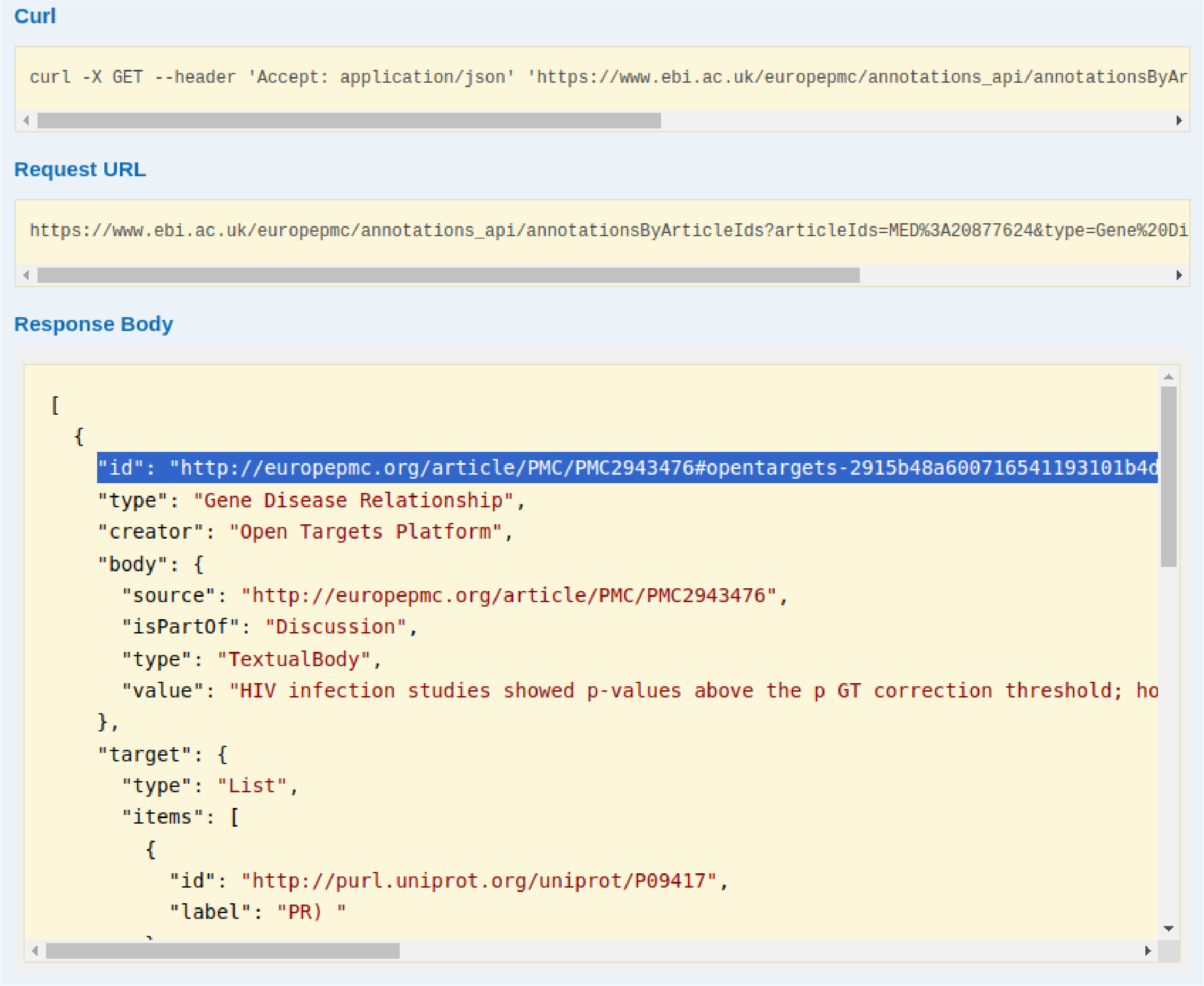

Fig. F2. The screenshot of the Open Target annotation (in JSON-LD format) retrieved from the Europe PMC Annotations API for the article with PMID 20877624. The annotation URI for the Open Target annotation is highlighted.

[14] `https://europepmc.github.io/techblog/algorithm/2018/07/04/locating-text-html-pages.html`

C. Making annotations FAIR and Citable. One of the main aims of establishing Europe PMC Annotation API is to make text-mined annotations FAIR, where users are able cite the text-mined entities and relationships. This allows the consumer of the content to understand and trace the source of information. The Web Annotation Model specification allows annotations to be uniquely identified using URIs, offering a mechanism to cite annotations across multiple Platforms. To this end, for all Open Targets annotations we have minted resolvable annotation URIs. For instance, for a given article ID the corresponding Open Target annotation can be retrieved in JSON-LD format, that will contain the annotation URI (see Figure F2).

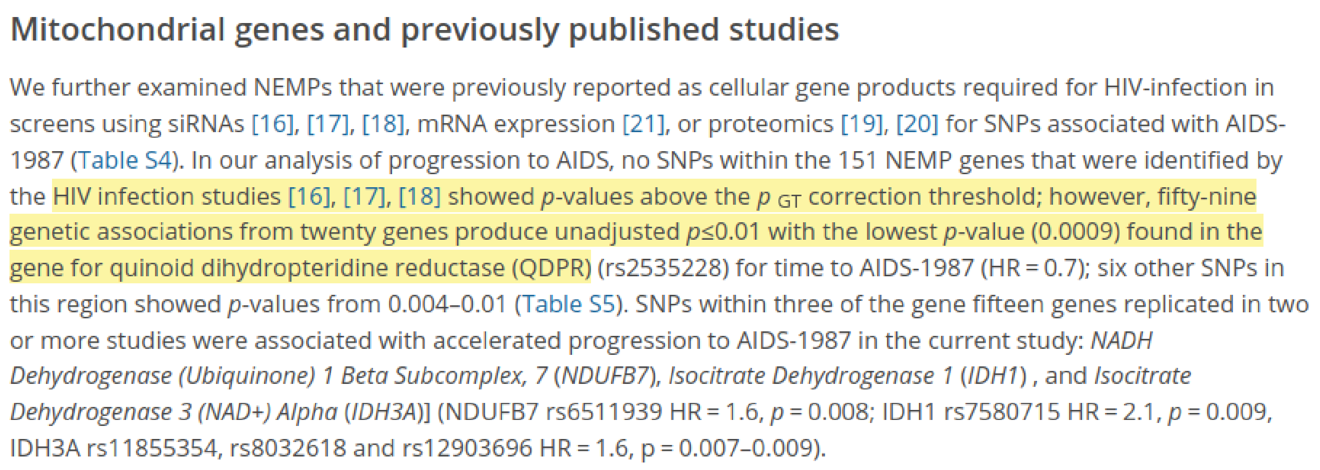

Mitochondrial genes and previously published studies

We further examined NEMPs that were previously reported as cellular gene products required for HIV-infection in screens using siRNAs [16], [17], [18], mRNA expression [21], or proteomics [19], [20] for SNPs associated with AIDS-1987 (Table S4). In our analysis of progression to AIDS, no SNPs within the 151 NEMP genes that were identified by the HIV infection studies [16], [17], [18] showed *p*-values above the p_{GT} correction threshold; however, fifty-nine genetic associations from twenty genes produce unadjusted $p \leq 0.01$ with the lowest *p*-value (0.0009) found in the gene for quinoid dihydropteridine reductase (QDPR) (rs2535228) for time to AIDS-1987 (HR = 0.7); six other SNPs in this region showed *p*-values from 0.004–0.01 (Table S5). SNPs within three of the gene fifteen genes replicated in two or more studies were associated with accelerated progression to AIDS-1987 in the current study: *NADH Dehydrogenase (Ubiquinone) 1 Beta Subcomplex, 7* (*NDUFB7*), *Isocitrate Dehydrogenase 1* (*IDH1*) , and *Isocitrate Dehydrogenase 3 (NAD+) Alpha* (*IDH3A*)] (NDUFB7 rs6511939 HR = 1.6, *p* = 0.008; IDH1 rs7580715 HR = 2.1, *p* = 0.009, IDH3A rs11855354, rs8032618 and rs12903696 HR = 1.6, p = 0.007–0.009).

Fig. F3. SciLite annotation tool highlighting the gene-disease association between HIV infection and QDPR using the LinkBack call. The LinkBack feature is based on the LinkBack API (which accepts the unique 'code' in an annotation ID) and text-annotator.

D. Results in the Open Targets Platform. The Europe PMC dataset is utilised in two ways: The first is to use the dataset to extract evidence for the association of targets and diseases. Co-occurrences of target and disease entities are considered evidence for the association of those entities. In detail, when a target and a disease are mentioned in the same sentence within a publication, this constitutes one piece of Europe PMC evidence for the association of that target and that disease [Figure F4 (a)]. The second involves using the dataset to provide context to the Platform entities. Users can browse the available literature for the entity of their choice through the Bibliography widget, for example all the papers linked to cystic fibrosis [Figure F4(b)]. For more details refer to article (2, 12).

S8: Comparison with Other Tools

The outputs from the lit-OTAR framework are visualised and accessed through Europe PMC's Scilite/Annotations API and Open Targets Platform. Table T4 present comparison of various tools including SemMedDB (8), LitSense (9), PubTator (10) and PubTator Central (11) which provide similar text-mining outputs.

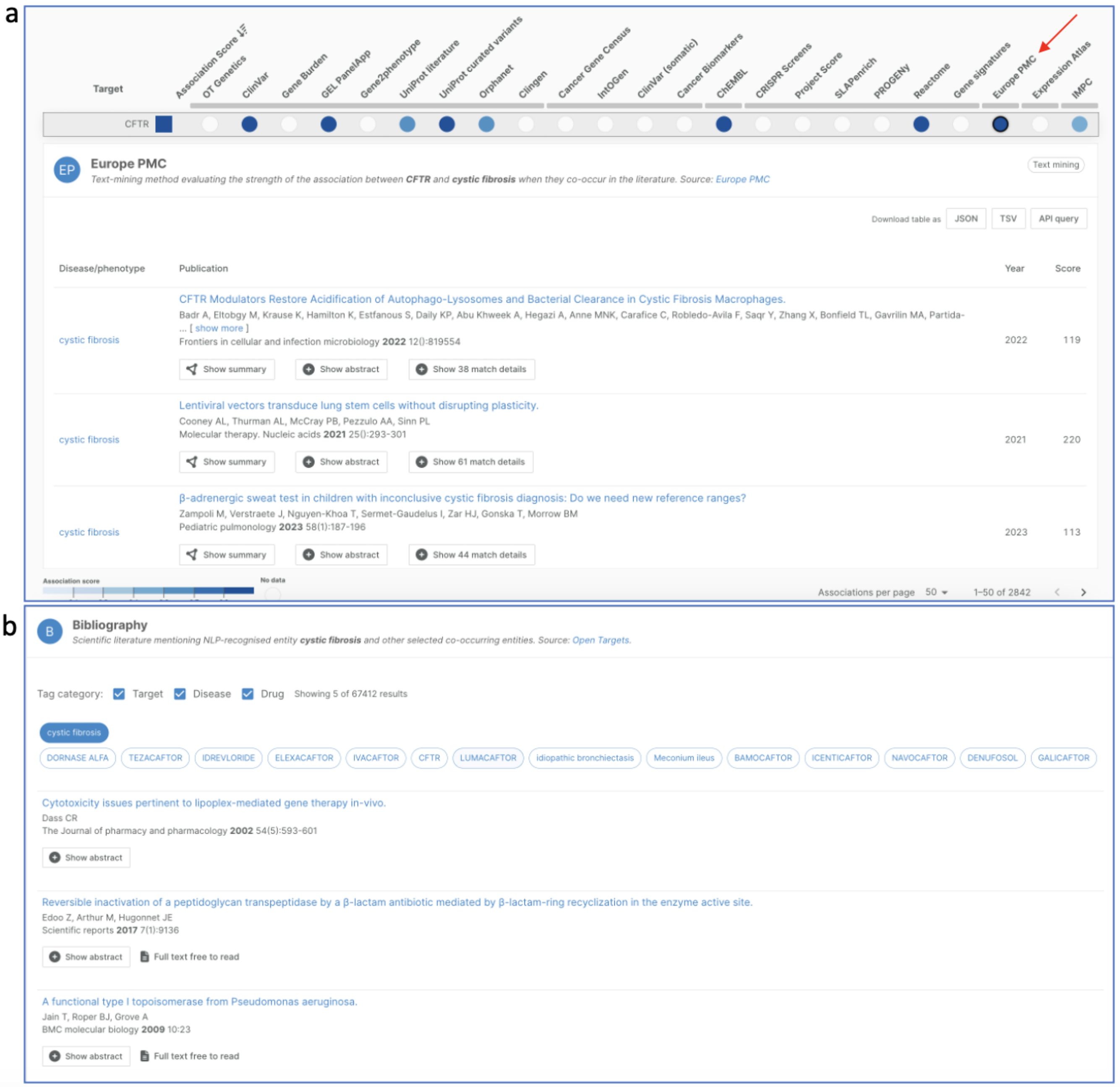

Fig. F4. Summary of how the lit-OTAR results are utilised and visualised in the Open Targets Platform. a. Europe PMC (red arrow) as data source for evidence of target–disease associations; b. Bibliography widget from a disease profile page.

Feature	SciLite/Annotations API (3)	LitSense (9)	SemMedDB (8)	PubTator 3.0 (10, 11)
Developer	Europe PMC	NCBI	NLM (National Library of Medicine)	NCBI
Primary Function	Display text-mined annotations to link articles with biological data	Sentence-level retrieval of biomedical literature	Semantic predications extraction and summarization from biomedical text	text-mining, entity annotation, and relation extraction
Entity Types Annotated	Primarily Gene/protein names, diseases, organisms, chemicals, gene ontology terms, experimental methods, Accesion numbers, Resources. Many other entities from other providers	Genes, proteins, diseases, chemicals, mutations, species	Entities include UMLS concepts (e.g., drugs, diseases, genes, anatomy, etc.)	Genes, diseases, chemicals, variant, species, cellline
Relations Extracted	Gene–disease, protein–protein interactions, transcription factor–gene targets, and biological events.	None	Subject–predicate–object triples (e.g., TREATS, AFFECTS, PROCESS_OF, etc.)	33 million relations (8.8 million unique pairs)
Scale of Data	Integrates multiple text-mining tools, e.g., ExTRI, IntAct, DisGeNET, PheneBank, Open Targets, and OntoGene, Metagenomics. More than 2 billion annotations.	Focused on sentence-level data	Database includes detailed structured data: citations, sentences, entities, and coreferences	1.6 billion entity annotations (4.6 million unique identifiers)
Data Sources	Europe PMC articles and curated data sources (e.g., ExTRI, IntAct, Open Targets, DisGeNET)	PubMed abstracts and PMC full-text articles	PubMed abstracts; entities mapped to UMLS Metathesaurus concepts	PubMed abstracts and PMC full-text articles
User Interface	Highlights terms within articles and links them to external databases and tools. Exclusive API access with search	Displays relevant sentences with highlighted entities	Database schema available for querying; detailed auxiliary and semantic data accessible	Web interface and API with search
Update Frequency	Daily regular updates with Europe PMC content	Regular updates with PubMed and PMC content	Periodic updates; schema and data aligned with the latest biomedical literature	Weekly updates from PubMed and PMC
Customization	Users can select specific annotation types to display (e.g., gene-disease, protein interactions)	Users can filter results by article section or publication year	Supports custom queries on predications, coreferences, and auxiliary data	Supports semantic and relational queries with enhanced precision
Integration	Integrated within Europe PMC platform and connects with text-mining tools	Integrated with PubTator for entity highlighting	Can be integrated into other systems via its detailed relational schema	Integrated with NCBI resources like PubMed and PMC
Community	Users can upload their own data to support community	None	None	None
Performance	Enhanced linking of literature to biological data; focuses on annotation coverage rather than precision	Efficient for sentence-level searches	Proven semantic predication quality and flexibility in querying relationships	Relation extraction and search precision in top 20 results

Table T4. Comparison of various biomedical scientific literature tools providing gene-drug-disease annotations.

DRAFT
